# Profile of cognitive impairment in late‐stage Parkinson's disease

**DOI:** 10.1002/brb3.2537

**Published:** 2022-03-07

**Authors:** Catarina Severiano e Sousa, Margherita Fabbri, Catarina Godinho, Rita Moiron Simões, Inês Chendo, Miguel Coelho, Isabel Pavão Martins, Joaquim J. Ferreira

**Affiliations:** ^1^ Laboratory of Clinical Pharmacology and Therapeutics Faculdade de Medicina Universidade de Lisboa Lisboa Portugal; ^2^ Faculdade de Medicina Instituto de Medicina Molecular João Lobo Antunes Universidade de Lisboa Lisboa Portugal; ^3^ Department of Neurosciences Clinical investigation Center CIC 1436, Parkinson Toulouse Expert Center, NS‐Park/FCRIN Network and NeuroToul COEN Center Toulouse University Hospital INSERM University of Toulouse 3 Toulouse France; ^4^ Grupo de Patologia Médica Nutrição e Exercício Clínico (PaMNEC) do Centro de Investigação Interdisciplinar Egas Moniz (CiiEM) Escola Superior de Saúde Egas Moniz Almada Portugal; ^5^ Neurology Department Hospital Beatriz Ângelo Loures Portugal; ^6^ Clínica Universitária de Psiquiatria Faculdade de Medicina Universidade de Lisboa Lisbon Portugal; ^7^ Psychiatry Department Department of Neurosciences Hospital de Santa Maria Lisbon Portugal; ^8^ Neurology Service Department of Neurosciences Hospital Santa Maria Lisbon Portugal; ^9^ Laboratório de Estudos de Linguagem Faculdade de Medicina Universidade de Lisboa Lisbon Portugal; ^10^ Campus Neurológico Torres Vedras Portugal

**Keywords:** cognitive impairment, late‐stage, Parkinson's disease

## Abstract

**Introduction:**

The profile of cognitive impairment associated with the late stages of Parkinson's disease (LSPD) is rarely reported. Its characterization is necessary to better understand the cognitive changes that occur as the disease progresses and to better contribute to its management.

**Methods:**

In this cross‐sectional study, we characterized the cognitive profile of LSPD patients using the comprehensive assessment methodology proposed by the International Parkinson and Movement Disorders Society Task Force. The association of clinical and demographic variables with dementia diagnosis was also investigated using binary logistic regression analysis.

**Results:**

Eighty‐four LSPD patients were included (age 75.4 ± 6.9; disease duration 16.9 ± 7.5). Fifty‐four (64.3%) were classified as demented and presented a global impairment cognitive profile. In the nondemented group (*N* = 30), 25 (83.3%) LSPD patients met the diagnostic criteria for mild cognitive impairment, mostly with multiple domain impairment (96.0%) and a heterogeneous profile. Memory was the most frequent and severely impaired cognitive domain in both groups. Disease disability, orientation, complex order comprehension, verbal learning, and visuoconstructive abilities were significantly associated with dementia diagnosis (*p* < .05).

**Conclusions:**

Cognitive impairment in multiple domains was common in LSPD patients. The most frequent and prominent deficits were in the memory domain, with a strong interference from attention impairment. Disease disability, orientation, complex order comprehension, verbal learning, and visuoconstructive abilities proved to be important determinants for dementia diagnosis.

## Graphical abstract

We have characterized the cognitive profile of 84 LSPD patients. Almost all LSPD patients presented cognitive impairment. Fifty‐four LSPD patients (64%) were classified as demented, most of them presented a global impairment profile, with both Alzheimer's and PD‐typical impairments. Twenty‐five patients (30%) presented mild cognitive impairment, mainly with a heterogeneous profile. Disease disability, orientation, complex order comprehension, verbal learning, and visuoconstruction were found to contribute to the dementia diagnosis.

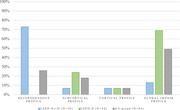

## 1 INTRODUCTION

Cognitive dysfunction is one of the most prevalent nonmotor symptoms associated with Parkinson's disease (PD) and has been widely studied in an attempt to better describe the pattern of impairment and the cognitive profile of PD patients. However, there are few published studies on late‐stage Parkinson's disease (LSPD). Indeed, LSPD is a poorly studied population due to the typically severe motor and nonmotor symptoms that often complicate cognitive assessment. This may be the reason why studies including this population are neuropathological studies (Braak et al., 2005), assess cognition using cognitive screening tests (Coelho et al., 2015), or do not make a detailed description of cognitive performance (Reid et al., 2011). It is common to observe the inclusion of this population in heterogeneous groups regarding the motor dysfunction/severity associated with PD, without providing a particular description of their cognitive profile.

Dementia has been described as a nonmotor symptom that is almost inevitable, mainly in the advanced stages of PD (Reid et al., 2011). It is a crucial determinant of reduced life expectancy in patients with PD. Defining the pattern of the cognitive impairment that is prodromal of Parkinson's disease dementia (PDD) is an area of active research owing to its predictive value and the possibility that these deficits could respond better to treatments used in dementia (Kehagia et al., 2010).

Therefore, a greater understanding of the cognitive disorders present in LSPD is of the utmost importance due to their high frequency in these patients. In the same way, studying cognition in LSPD will help to better understand the pattern of cognitive impairment across the different PD stages.

The current study sought to help clarify these issues by examining the cognitive performance of LSPD patients. To assess dementia we followed the methodology suggested by MDS PDD Level II (Dubois et al., 2007a) and examined cognitive profiles according to the pattern of cognitive impairment. We also investigated clinical, demographic, and cognitive variables that are associated with PDD diagnosis.

## 2 METHODS

### 2.1 Subjects

In this cross‐sectional study, consecutive LSPD patients attending the Movement Disorders Unit of the University Hospital of Santa Maria (Lisbon) were recruited.

All participants and their formal/informal caregivers were informed about the study objectives and procedures and were asked for their written informed consent. The informed consent of cognitively impaired patients was signed by the caregiver. The study was approved by the local ethics committee.

The inclusion criteria were (1) idiopathic PD according to the UK Brain Bank criteria (Hughes et al., 1992); (2) late‐stage Parkinson's disease (Coelho & Ferreira, 2012) (Hoehn and Yahr scale [H&Y] score > 3 [Goetz et al., 2004] and a Schwab and England scale [S&E] score < 50% [Schwab & England, 1969] in the ON condition), and (3) signed informed consent. Patients were excluded if they presented dementia before PD onset or dementia within 1 year of PD diagnosis.

### 2.2 Measures

#### 2.2.1 Neurologic and functional assessment

Neurological assessment was performed by a neurologist with expertise in movement disorders and included the investigation of vascular problems. It took place at the the Movement Disorders Unit of the University Hospital of Santa Maria or, whenever this was not possible (due to difficulties in patients’ mobility), it took place at patients’ home. The Movement Disorders Society—Unified Parkinson Disease Rating Scale (MDS–UPDRS) (Parts I and IV) (Goetz et al., 2008) was administered to assess PD motor and nonmotor features. We specified clarity of handwriting and tremor item results (Part II 2.7 and 2.10 items) to better analyse the results, particularly of paper and pencil cognitive tests. The neuropsychiatric functions were assessed by the same neurologist and included behavioral symptoms and major depression, using the neuropsychiatric inventory (Cummings et al., 1994) (NPI) and the geriatric depression scale (Ertan et al., 2005) (GDS), respectively. *Delirium* was diagnosed according to the DSM‐5 criteria (American Psychiatric Association, 2014).

#### 2.2.2 Neuropsychological assessment

The neuropsychological assessment (NPA) was performed by a neuropsychologist. It took place in a single session, at the patients' homes, to ensure the presence of the usual amenities and to reduce as far as possible any interference in the patients’ lives.

A clinical interview was undertaken, with caregiver collaboration, to obtain each LSPD patient's sociodemographic and clinical information.

To assess the impact of cognitive impairment on activities of daily living (ADL), two approaches were taken: 1) a patient and caregiver clinical interview and 2) the Pill Questionnaire (Dubois et al., 2007a). Whenever one of these sources of information was suggestive of dysfunction, the patient was classified as having impaired ADL.

The LSPD patient´s cognitive performance was described by using a neuropsychological battery which was applied in the same order for all patients and included a set of cognitive tests selected from the MDS PDD Level II proposal (Table 1), according to the following criteria: 1) existence of normative data for the Portuguese population and 2) expected time consumed by the test (only one test for each cognitive task whose performance would have a shorter estimated duration, to minimise interfering with tiredness). According to the MDS Level II recommendations, the memory domain was dissociated into two components to better understand the pattern of its impairment: 1) subcortical‐frontal component (stategic aspects of explicit memory) and 2) mediotemporal component (encoding and retrieval abilities).

**TABLE 1:** Neuropsychological tests used to assess cognition according to MDS PDD Level II criteria (Dubois et al., 2007a)

Cognitive domains/tasks	MDS PDD Level IICognitive tests
Global efficiency	MMSE (Folstein et al., 1975; Morgado et al., 2009)
Executive functions	
Working memory	Digit span (Martins et al., 2013; Wechsler, 1997a)
Conceptualization	Similarities (WAIS‐III) (Wechsler, 1997b; Wechsler, 2008
Set activation	Phonologic fluency (P,M,R) (Benton & Hamsher, 1989; Cavaco et al., 2013a)
Set shifting	TMT (A and B) (Cavaco et al., 2013b; Reitan, 1958)
Set maintenance	Odd man out test (Flowers & Robertson, 1985)
Behavioral control	Prehension behavior (FAB) (Lhermitte et al., 1986; Lima et al., 2008)
Memory	RAVLT (Cavaco et al., 2015; Schmidt, 1996)
Instrumental functions	
Language	Boston naming test (Kaplan et al., 1983; Peña‐Casanova et al., 2009)
Visuoconstructive	Copy of the clock (Sunderland et al., 1989)
Visuospatial	Benton line orientation test (Benton et al., 1994)
Visuoperceptive	Benton face recognition test (Benton et al., 1994)

MDS PDD, Parkinson's disease dementia criteria recommended by Movement Disorder Society Task Force; MMSE, Mini‐Mental State Exam; TMT (A and B), trail making test A and B; WAIS‐III, Wechsler Adult Intelligence Scale, 3rd edition; FAB, frontal assessment battery; RAVLT, Rey auditory and verbal learning test.

Raw scores for cognitive tests were transformed into *z*‐scores based upon normative data for the Portuguese population and upon manual tests, which were adjusted for age, education, and, when possible, for sex. Each LSPD patient's cognitive performance was obtained by composite scores for each cognitive domain, which were calculated by averaging individual cognitive *z*‐scores within each of the specific domains.

We considered a cognitive test/domain as impaired if its *z*‐score was more than 1 standard deviation (SD) below the adjusted normative scores (*z*‐score ← 1).

The cognitive profile was described based on the organization of cognitive domains proposed by the MDS PDD Level II (Dubois et al., 2007a) and the respective pattern of cognitive impairment: (1) subcortical profile that included patients with executive function impairment and only subcortical‐frontal memory component impairment and 2) cortical profile that included patients with instrumental function impairment and only in the mediotemporal memory component. The patients who did not present, or presented changes in the cognitive domains that did not fulfill a specific cognitive profile, were classified as having a heterogeneous profile. The patients who manifested changes in both subcortical and cortical mediated cognitive domains were classified as having a global impairment profile. The LSPD patient´s cognitive profile was drawn through composite scores which were obtained by averaging the cognitive domains z‐score within each of the specific cognitive profiles.

### 2.3 Cognitive categorization

LSPD patients were classified as demented or nondemented. Dementia was diagnosed according to the MDS PDD Level II criteria (Dubois et al., 2007a) based on neuropsychological and functional autonomy assessment and a clinical interview.

For exploratory purposes, we examined the frequency of mild cognitive impairment (MCI) by using the MDS MCI Level II criteria (comprehensive assessment) (Litvan et al., 2012).

### 2.4 Statistical analysis

To describe the cognitive performance of LSPD patients, we compared clinical and demographic variables, and neuropsychological z‐scores of the demented and nondemented patients by using the Chi‐square, Mann‐Whitney U, and Wilcoxon tests, as appropriate (*p* < .05).

Subsequently, we analysed cognitive profile subgroups by comparing clinical and cognitive variables in each of the demented and nondemented groups and between groups, using Chi‐square, Wilcoxon, Mann‐Whitney U, and Kruskal‐Wallis tests with post‐hoc pairwise analysis with Bonferroni correction of (*p* < .05), as appropriate.

Finally, to examine the contribution of the clinical, demographic and neuropsychological measures for dementia diagnosis, binary logistic regression analysis with a forward conditional procedure was run with all of the variables that reached statistical significance (*p* < .05) between demented and nondemented groups as independent variables and with presence or absence of dementia as the dependent variable. Per block step, the improvement of the regression model was compared using the likelihood ratio test. The goodness of fit of the model was assessed with the Hosmer‐Lemeshow test.

All statistical analyses were performed using SPSS software version 26 (IBM SPSS, Chicago, IL).

## 3 RESULTS

### 3.1 Patient demographics and clinical characteristics

Eighty‐four LSPD patients performed the NPA. Forty‐eight (57.1%) were women, with a mean age of 75.4 years (±6.9), 6.5 years (±4.5) of education, 16.9 years (±7.5) of disease duration, and 58.5 years (±10.9) at PD onset. 97.3% were on levodopa treatment (Table 2).

**TABLE 2:** Demographic and clinical characteristics of LSPD patients

	LSPD (*N* = 84) Mean (SD)	LSPD‐NoD (*N* = 30) Mean (SD)	LSPD‐D (*N* = 54) Mean (SD)	*p*
Gender (M/F)	36/49	9/21	27/27	.08
Age (years)	75.4 (6.9)	75.8 (5.2)	75.2 (7.7)	.92
Education (years)	6.5 (4.5)	5.7 (4.4)	7.1 (4.5)	.14
Disease duration (years)	16.9 (7.5)	16.9 (7.7)	16.9 (7.6)	.86
Age at onset (years)	58.5 (10.9)	58.9 (10.2)	58.2 (11.2)	.94
MDS–UPDRS Part I	20.6(6.9)	17.1(6.4)	22.6(6.4)	.00
MDS–UPDRS Part II	33.6(9.5)	29.9(9.0)	35.9(9.3)	.00
MDS–UPDRS Part II 2.7 (clarity of handwriting)	3.3 (0.9)	3.0(0.9)	3.4(0.9)	.02
MDS–UPDRS Part II 2.10 (tremor)	1.0 (1.2)	1.1 (1.1)	1.0 (1.2)	.72
MDS–UPDRS Part III	58.9 (16.6)	52.2 (15.4)	63.1 (16.1)	.00
MDS–UPDRS Part IV	5.1 (4.4)	4.8 (4.6)	5.1 (4.2)	.74
Levodopa (% yes)	97.3	100	95.8	.29
Hoehn & Yahr stage (0/5)	4.1 (0.9)	3.8 (0.9)	4.2 (0.9)	.03
Schwab & England (0/100%)	36.7 (13.6)	44.7 (14.8)	32.2 (10.7)	.00
NPI delusions (0/12) (cutoff ≥3)	1.3 (2.6)	0.6 (2.0)	1.8 (2.8)	.00
NPI hallucinations (0/12) (cutoff ≥3)	1.7 (2.7)	0.8 (2.2)	2.2 (2.9)	.01
NPI depression (0/12) (cutoff ≥3)	3.3 (2.4)	2.9 (2.5)	3.6 (2.3)	.13
NPI apathy (0/12) (cutoff ≥3)	3.6 (3.6)	2.2 (3.1)	4.5 (3.6)	.00
NPI sleep disorders (0/12) (cutoff ≥3)	2.5 (3.3)	1.9 (2.6)	2.9 (3.6)	.47
Pill questionnaire (0/3) (cutoff ≥2)	2.2 (1.1)	1.5 (1.1)	2.6(0.8)	.00
GDS score (0/30) (cutoff 11–20: mild depression; 21–30: severe depression)	13.8 (6.8)	13.9 (5.3)	17.0 (5.9)	.03
MMSE score (0/30) (cutoff: 0–2 years education: 22 pts; 3–6 years education: 24 pts; ≥7 years education: 27 pts)	21.6 (6.1)	27.1 (2.0)	18.4 (5.3)	.00

*Note*: Subgroups were compared using chi‐squared (nominal variables) and Mann–Whitney *U* tests (continuous variables) (*p* < .05 is significant).

LSPD, late‐stage Parkinson's disease; LSPD‐NoD, late‐stage Parkinson's disease patients without dementia; LSPD‐D, late‐stage Parkinson's disease patients with dementia; MDS–UPDRS, Movement Disorder Society Unified Parkinson's Disease Rating Scale; NPI, neuropsychiatric inventory; GDS, Geriatric Depression Scale; MMSE, Mini‐Mental State Exam.

According to MDS PDD Level II criteria, 54 (64.3%) LSPD patients met the criteria for PDD (27/36 men (75%), 27/49 women (55%)).

There were no statistically significant differences between demented and nondemented groups concerning sex, age, education, disease duration, age at PD onset, or levodopa treatment profile. We highlight the fact that there were no significant differences in the MDS–UPDRS tremor item, which arose in both groups with sufficiently low frequency or intensity to cause no impact on function, as well as in motor complications (MDS–UPDRS Part IV), whose frequency and severity were between mild and moderate in both groups (Table 2).

### 3.2 Cognitive performance of LSPD patients

In the nondemented group (*N* = 30), 5 (16.7%) LSPD patients did not present changes in cognitive domains. The remaining 25 (83.3%) had impairment on at least two neuropsychological tests, meeting criteria for the diagnosis of MCI (4.0% with single‐domain impairment and 96.0% with multiple domain impairment).

**FIGURE 1:**
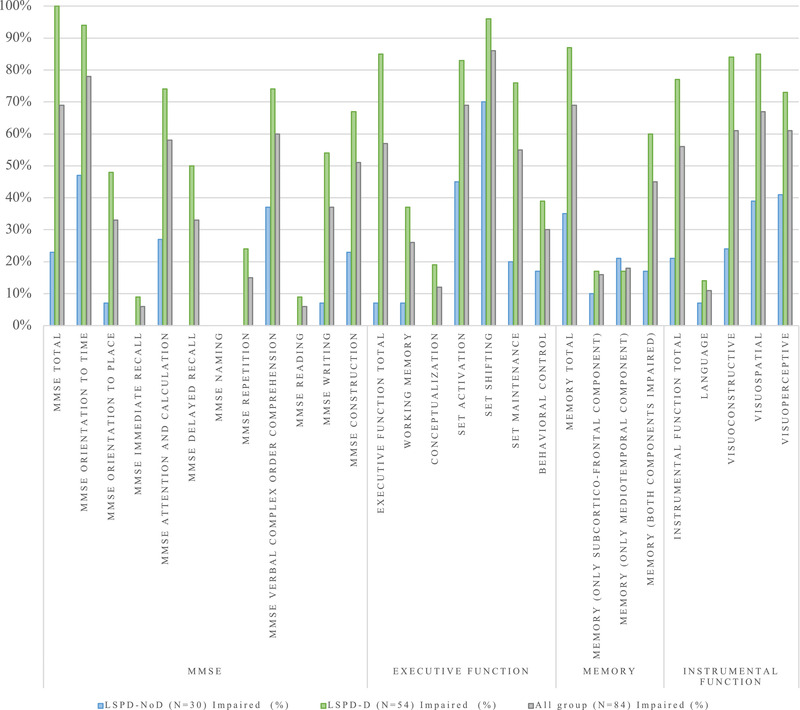
Frequency of cognitive impairment in LSPD patients. MMSE, Mini‐Mental State Exam; LSPD‐NoD, late‐stage Parkinson's disease patients without dementia; LSPD‐D, late‐stage Parkinson's disease patients with dementia

**FIGURE 2:**
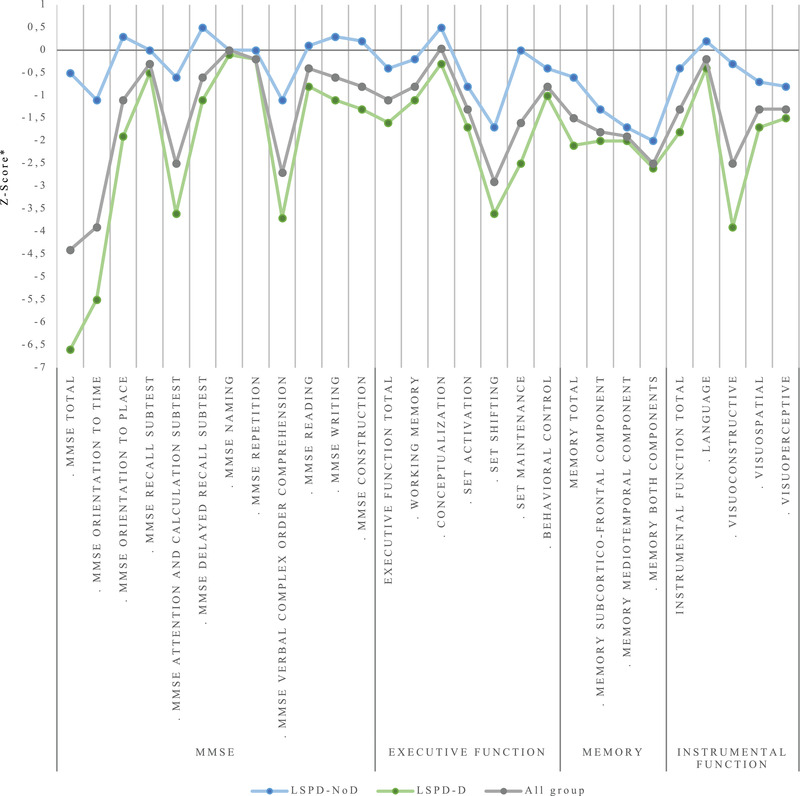
Cognitive performance of LSPD patients. MMSE, Mini‐Mental State Exam; LSPD‐NoD, late‐stage Parkinson's disease patients without dementia; LSPD‐D, late‐stage Parkinson's disease patients with dementia. *We considered a cognitive test/domain as impaired if its z‐score was more than 1 standard deviation below the adjusted normative scores (*z*‐score ← 1)

Memory emerged as the most frequently impaired cognitive domain (34.5% of the nondemented group) (Figure 1). When dissociating memory into its two components to analyze the memory deficit pattern, we observed that the mediotemporal component was the most frequently affected (20.7%), with an accentuated impairment (mean *z*‐score −1.7±0.5) (Figure 2) particularly in the long‐term percent retention. We also observed a high frequency of intrusions in immediate and delayed retrieval (60.0% with more than five intrusions).

Instrumental function was also impaired in nondemented LSPD (LSPD‐NoD) patients (20.7%), mainly visuoperceptive (40.7%) and visuospatial (39.3%) functions. Executive function was the least impaired cognitive domain in this group (6.7%). However, when we analyzed it in detail, we observed a high frequency of impairment in attention tasks, mainly in set shifting (70.4%) and set activation (44.8%).

In the demented group (*N* = 54), 40.7% of LSPD patients reported changes in multiple domains and 59.3% in all cognitive domains.

The memory cognitive domain was the most frequently impaired (86.8%). We dissociated it into its two components to analyze its deficit patterns, observing that the majority of demented LSPD (LSPD‐D) patients (60.4%) presented changes in both components, and that impairment was accentuated (mean z‐score −2.6±0.7), with a high frequency of intrusions (73.6% LSPD‐D patients with more than five intrusions).

For executive function, the second most impaired LSPD‐D cognitive domain (85.2%), we observed a pattern of changes similar to those in the nondemented group (mainly affecting attention) but with a much more pronounced frequency and impairment severity in all executive function cognitive tasks (*p* < .05).

Also, the instrumental function cognitive domain was significantly more impaired in the demented group (*p* = .00). When we looked at its pattern of changes, visuospatial was the function that was most frequently impaired (85.1%), followed by visuoconstructive (83.7%), two cognitive functions where there was also the most pronounced impairment (mean z‐score −3.9±2.6) (*p* < .05) (Table S1, supplementary material).

### 3.3 Cognitive profile of LSPD according to the pattern of cognitive impairment

The LSPD patients were classified according to their cognitive profile, based on the pattern of cognitive impairment.

**FIGURE 3:**
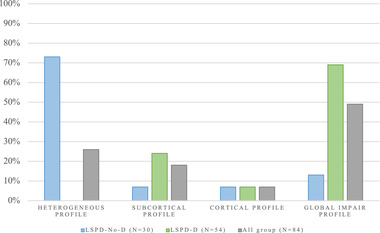
Frequency of cognitive profile of LSPD patients. LSPD‐NoD, late‐stage Parkinson's disease patients without dementia; LSPD‐D, late‐stage Parkinson's disease patients with dementia

As required by definition, most of the nondemented group (73.3%) did not present significant changes in the cognitive tasks underlying each cognitive profile. They presented a heterogeneous cognitive profile. Only 6.7% of LSPD patients were classified as having a subcortical profile, 6.7% as having a cortical profile and, 13.3% as having a global impairment profile (Figure 3).

In the demented group, 24.1% of LSPD patients were classified as having a subcortical profile, 7.4% as having a cortical profile, and 68.5% as having a global impairment profile.

As the cognitive profile subgroups were small, no statistically significant differences were found within and between the nondemented and demented groups regarding demographic, clinical, and most of the distribution of cognitive variables distribution (Table S2, supplementary material).

### 3.4 Variables associated with PDD

The hierarchical logistic regression analysis, with presence/absence of PDD as the dependent variable and clinical and neuropsychological measures as independent variables, showed that (1) the MMSE subscales scores of orientation in time (OR = 0.54, β = −0.63; 95% confidence interval [CI] = 0.35–0.83; *p* = .00), orientation in space (OR = 0.35, β = −1.04; 95% CI = 0.15–0.86; *p* = .02), language (complex order comprehension) (OR = 0.61, β = −0.50; 95% CI = 0.43–0.87; *p* = .00), and construction (OR = 0.48, β = −0.73; 95% CI = 0.25–0.93; *p* = .03), (2) S&E disability scale (OR = 0.91, β = −0.09; 95% CI = 0.85–0.98; *p* = .00), (3) RAVLT verbal learning (OR = 0.33, β = −1.11; 95% CI = 0.12–0.91; *p* = .03), and (4) copy of the clock test (OR = 0.62, β = −0.47; 95% CI = 0.45–0.87; *p* = .00) were associated with the condition of dementia, with reduced scores supporting the dementia diagnosis.

## 4 DISCUSSION

Our results indicate that 54 (64.3%) LSPD patients met the criteria for PDD. Of the 30 (35.7%) LSPD‐NoD patients, 5 (16.7%) did not present cognitive impairment. The remaining 25 (83.3%) met criteria for the diagnosis of MCI (4.0% with single‐domain impairment and 96.0% with multiple‐domain impairment). Reid et al. (2011) investigated a sample of PD who had 20 years of disease and found that 80% of PD patients had dementia. Based on these results, we expected to find a higher frequency of PDD. This discrepancy may be due in part to some clinical and sociodemographic characteristics of our sample. Phongpreecha et al. (2020) studied the associations between biological, clinical, and cognitive factors and they found that the primary factors associated with PDD were male sex, the glucocerebrosidase gene, later age at PD onset, and disease duration. In our study, although there were no statistically significant differences between the groups of demented and nondemented patients with regard to sociodemographic variables (probably due to the small size of the nondemented group, which will have reduced power of statistical analysis), our sample has an earlier age at PD onset and a higher proportion of women (58%) than expected based on previous reports. On the other hand, the participants had a level of education (mean of 6 years) that is not representative of the elderly Portuguese population who is poorly educated (up to 4 years in the population over 65 years), a fact that may have contributed to the lower frequency of dementia found.

Analysis of the cognitive performance of LSPD patients indicated that most of the demented and nondemented patients presented impairment in multiple cognitive domains.

Memory was the cognitive domain most frequently impaired, and the severity of the changes was also the most pronounced in both groups. Analysis by components showed that in the nondemented group, impairment prevailed in the mediotemporal component, with more frequent changes in long‐term retention, with a high frequency of intrusions in immediate and differed evocation, which also reveals that LSPD‐NoD patients have difficulty maintaining the distinction between information coming from the outside and their own associations (Lezak et al., 2012). These results are supported by some studies that report that the performance of PD patients in explicit memory tests is significantly decreased in those tasks that require the organization of the to‐be‐remembered material (Buytenhuijs et al., 1994). Internal control of attention, required to generate spontaneously efficient encoding and retrieval strategies, is impaired in PD (Dubois & Pillon, 1997). Recall deficit is not primarily due to a memory disruption but rather to difficulties in activating the neuronal processes involved in the functional use of memory stores (Dubois & Pillon, 1997). PD patients are not unable to acquire mental sets but have difficulty maintaining newly acquired sets against competing alternatives, suggesting again a deficit of internal control of attention (Dubois & Pillon, 1997).

In the demented group, we observed a global memory impairment with changes both in learning and retention, and there was a high frequency of intrusions. These results revealed a global impairment in the different stages of information storage, whose severity was much more accentuated than that manifested by the nondemented group.

For instrumental function, the visuoperceptive abilities were the most frequently impaired in nondemented patients. Some neuroimaging studies report that these changes often arise in association with posterior cortical dysfunction (Abe et al., 2003).

In the demented group, visuoconstructive and visuospatial functions were the most frequently impaired. The LSPD‐D patients correctly identified the clock design but manifested great difficulty in copying it, thereby revealing a visuoconstructive apraxia. PDD patients may be at an enhanced risk of visuospatial impairments that also reflect disrupted praxis and disrupted visuoperception over and above the contributions of motor dysfunction (Troster, 2008).

We highlight the low frequency of language changes (in both groups), assessed by the Boston Naming Test. Errors on this test, although infrequent, sometimes arose due to visuoperceptive changes and not due to an impairment in naming. These findings go against what is reported in the literature. Naming is not frequently reported as an impaired cognitive task in PD (Cummings et al., 1988).

Executive function, the cognitive domain often reported as most frequently impaired in PD, was the third most frequently impaired in the nondemented group and the second in the demented group. This discrepancy may be due to two reasons: (1) the decline in executive function occurs in the early stages and then tapers off during the mid‐stages of PD (Muslimovic et al., 2007) and (2) almost all LSPD patients were medicated with levodopa, which may improve executive function and attention (Lange et al., 1992). By contrast, visuospatial function (Lee et al., 1998), visual recognition memory, conditional associative learning, and verbal memory seem to be dopamine‐independent and unaffected by medication status (Owen et al., 1993; Sahakian et al., 1988). However, we highlight the changes we observed in attention both in frequency and severity of impairment. It was in the set shifting that LSPD patients manifested greater difficulties, even removing the speed element from the test evaluation. In the same way, in both groups, we observed changes in the set activation and the set maintenance. These changes may be attributed to the difficulty in maintaining and adapting a strategy against other competing possibilities due to frontal cortical and basal ganglia lesions (Flowers & Robertson, 1985).

When we grouped the cognitive domains into cognitive profiles to better characterize the substrate of cognitive impairment, we observed that the majority of LSPD‐NoD patients manifested a heterogeneous cognitive profile. Some authors suggest that this heterogeneity might be at least partly explained by uneven dopamine loss across the basal ganglia circuitry (Lewis & Barker, 2009; Sawamoto et al., 2008) and neurodegenerative hallmarks such as the emergence of cortical Lewy bodies and non‐Parkinson's disease features as a consequence of ageing (Kempster et al., 2010), which might interact with the putative pathological processes that underlies dementia. As such, there have been very few LSPD‐NoD patients in cognitive profile subgroups, which made it impossible for us to have a reliable comparative analysis between them.

In the demented group, most patients exhibited widespread cognitive deficits, which we classified as a global impairment cognitive profile. For this reason, once again, the comparative analysis with the remaining demented and nondemented subgroups proved delicate given their small size. For purely exploratory reasons, we observed that LSPD‐D patients in the global impairment group tended to be older, less educated, and have later PD age onset, greater disease disability, and overall greater frequency and severity of neuropsychiatric symptoms. It has been suggested that older age (Palazzini et al., 1995), later disease onset (after the age of 60 years) (Glatt et al., 1996; Reid, 1992), and low educational attainment (Glatt et al., 1996) are associated with greater cognitive decline. Our results went in this direction. When we compared them with the other demented cognitive profile subgroups, we found that they present, overall, greater impairment in cognitive domains.

Hobson and Meara (2004) in their elderly PD cohort (mean age of 78 years) ascribed memory deficits to the possible development of Alzheimer's disease (AD). Similarly, our elderly LSPD patients, mainly PDD patients with global cognitive impairment, appeared to present cognitive performance characterized by both AD (with memory [encoding] and orientation deficits) and PDD‐typical impairments (executive function, memory [retrieving], and visuospatial abilities). Although AD cannot be diagnosed in the presence of PD because, according to the National Institute of Neurological Disorders and Stroke‐Alzheimer Disease and Related Disorders (NINCDS‐ADRDA) criteria (Dubois et al., 2007b), early parkinsonian signs rule this diagnosis out, Bothe et al. (2010) maintain that these existing criteria are quite restrictive and they disregard the neuropathological findings of the Neuropathology Group of the Medical Research Council Cognitive Function and Ageing Study (2001), who mentioned that the majority of demented patients aged over 70 years present with more than one kind of severe neurodegenerative pathology.

A final finding of our study was that among the sociodemographic and clinical variables, only disease disability was significantly associated with PDD. Regarding the cognitive variables, we also found that orientation in time and space, complex order comprehension, and construction MMSE subscales revealed an important association with PDD. The same was observed with verbal learning and with the copy of the clock test. These findings are consistent with previous studies that reported associations between language, memory (Hobson & Meara, 2004), and visuoconstructive function impairment (Williams‐Gray et al., 2007) with PDD.

There are several strengthts in the current study. To the best of our knowledge, this is the first study that applies the MDS PDD criteria in a sample exclusively consisting of LSPD patients, examined in their homes with a complete neuropsychological assessment.

Our study has some limitations. First, we used a cross‐sectional design and, to conclude on the cognitive changes with disease progression, it would be of interest to have a prospective cohort and longitudinal data. Second, the inexistence of a nonlate‐stage control group did not allow us to compare the pattern of the cognitive impairment in a different stage of PD by using the same assessment methodology.

In conclusion, almost all nondemented patients meet clinical criteria for MCI with multiple‐domain impairment. Memory was the most frequently impaired cognitive domain, with a strong contibution of attention deficit. The cognitive profiles were heterogeneous, without any meaningful pattern over the cognitive domains. Most demented patients presented a global impairment profile, with both AD and PDD‐typical impairments. Disease disability, orientation, complex order comprehension, verbal learning, and visuoconstruction were associated with PDD diagnosis.

Future studies with larger LSPD cohorts will be important to conclude about the eventual protective effect of sociodemographic variables for LSPD dementia and their typical cognitive profiles.

## AUTHOR CONTRIBUTIONS

Catarina Severiano e Sousa: Conceptualization (equal), data extraction (lead), formal analysis (lead), funding acquisition, investigation (lead), methodology, project administration (equal), visualization, writing—original draft preparation. Margherita Fabbri: Data extraction, formal analysis, writing—review and editing. Catarina Godinho: Data extraction, formal analysis, writing—review and editing. Rita Moiron Simões: Data extraction, formal analysis, writing—review and editing. Inês Chendo: Writing—review and editing. Miguel Coelho: Writing—review and editing. Isabel Pavão Martins: Conceptualization (equal), methodology, supervision, writing—review and editing. Joaquim J. Ferreira: Conceptualization (equal), investigation, methodology (lead), project administration (equal), supervision (lead), writing—review, peer review (lead) and editing (lead).

## CONFLICT OF INTEREST AND FINANCIAL DISCLOSURES

Catarina Severiano e Sousa: No conflict of interest to report. No additional disclosures to report. Margherita Fabbri: No conflict of interest to report. No specific funding was received for this work. She has received consultancy fees from AbbVie. No additional disclosures to report. Catarina Godinho: No conflict of interest to report. No specific funding was received for this work. No additional disclosures to report. Rita Simões: no conflict of interest to report. No specific funding was received for this work. She has received consultancy fees from BIAL—PORTELA & Ca, SA, Zambon, and AbbVie. No additional disclosures to report. Inês Chendo: No conflict of interest to report. No specific funding was received for this work. No additional disclosures to report. Miguel Coelho: No conflict of interest to report. No specific funding was received for this work. He has received consultancy fees from Zambon. No additional disclosures to report. Isabel Pavão Martins: No conflict of interest to report. No specific funding was received for this work. No additional disclosures to report. Joaquim J. Ferreira: No conflict of interest to report. No specific funding was received for this work. He has received grants from GlaxoSmithKline, Grunenthal, Fundação MSD (Portugal), TEVA, MSD, Allergan, Novartis, Medtronic. He received consultancy fees from GlaxoSmithKline, Novartis, TEVA, Lundbeck, Solvay, BIAL, Merck‐Serono, Merz, Ipsen, Biogen, Acadia, Allergan, Abbvie, Sunovion Pharmaceuticals, Zambon, Affiris. He has also participated in advisory boards for Bial and provided expert testimony to Novartis.

## Supporting information

TABLE S1. Cognitive performance of LSPD patients

TABLE S2. Comparative analysis of the LSPD patients’ cognitive performance according to their cognitive profile in nondementia vs dementia groups

## Data Availability

The data that support the findings of this study are available in the supplementary material of this article.
