# Supplementary material for: Profile of cognitive impairment in late‐stage Parkinson's disease
Source: Brain Behav. 2022 Mar 7;12(4):e2537. doi: 10.1002/brb3.2537 (PMC9014988; doi:10.1002/brb3.2537)
Supplement: Supplementary file 1 — TABLE S1. Cognitive performance of LSPD patients [file BRB3-12-e2537-s001.docx]

| **Cognitive domains** | **Cognitive tests** | **LSPD**  **(N=84)** | | **LSPD-NoD**  **(N=30)** | | **LSPD-D**  **(N=54)** | |  |
| --- | --- | --- | --- | --- | --- | --- | --- | --- |
| MDS PDD  Level II domains |  | **Impaired**  **(%)** | **z-score Mean (SD)** | **Impaired**  **(%)** | **z-score Mean (SD)** | **Impaired**  **(%)** | **z-score Mean (SD)** | **p** |
| Global efficiency | . MMSE | 69.4 | -4.4(4.0) | 23.3 | -0.5(1.3) | 100 | -6.6(3.4) | **0.00** |
| 1. Orientation | . MMSE Orientation subtest | 61.2 | -3.3(3.6) | 20.0 | -0.4(1.2) | 85.2 | -4.9(3.5) | **0.00** |
| 1.1. orientation to time | . MMSE Orientation subtest | 77.6 | -3.9(3.7) | 46.7 | -1.1(2.0) | 94.4 | -5.5(3.6)**^f^** | **0.00** |
| 1.2. orientation to place | . MMSE Orientation subtest | 32.9 | -1.1(2.4) | 6.7 | 0.3(0.6) | 48.1 | -1.9(2.7) | **0.00** |
| 2. Immediate recall | . MMSE Recall subtest | 5.9 | -0.3(1.1) | 0 | 0.0(0.0) | 9.3 | -0.5(1.3) | **0.03** |
| 3. Attention and calculation | . MMSE Attention and calculation subtest | 57.6 | -2.5(4.0) | 26.7 | -0.6(1.5) | 74.1 | -3.6(4.5)**^d,e^** | **0.00** |
| 4. Delayed recall | . MMSE Delayed recall subtest | 32.9 | -0.6(1.3) | 0 | 0.5(0.6) | 50.0 | -1.1(1.2)**^d^** | **0.00** |
| 5. Language | . MMSE Language subtest | 58.8 | -2.6(3.1) | 20.0 | -0.3(1.3) | 81.5 | -3.8(3.2)**^d^** | **0.00** |
| 5.1. naming | . MMSE Language subtest | 0 | -0.0(0.2) | 0 | 0.0(0.0) | 0 | -0.1(0.2) | 0.20 |
| 5.2. repetition | . MMSE Language subtest | 15.3 | -0.2(0.4) | 0 | 0.0(0.0) | 24.1 | -0.2(0.4) | **0.00** |
| 5.3. verbal complex order comprehension | . MMSE Language subtest | 60.0 | -2.7(3.5) | 36.7 | -1.1(2.4) | 74.1 | -3.7(3.7)**^g^** | **0.00** |
| 5.4. reading comprehension | . MMSE Language subtest | 5.9 | -0.4(2.2) | 0 | 0.1(0.2) | 9.3 | -0.8(2.7) | 0.14 |
| 5.5. writing | . MMSE Language subtest | 36.5 | -0.6(1.5) | 6.7 | 0.3(0.6) | 53.7 | -1.1(1.6) | **0.00** |
| 6. Construction | . MMSE Construction subtest | 50.6 | -0.8(1.3) | 23.3 | 0.2(1.2) | 66.7 | -1.3(1.0)**^d^** | **0.00** |
| Subcortical features |  |  |  |  |  |  |  |  |
| 1. Executive function |  | 56.5 | -1.1(0.8) | 6.7 | -0.4(0.5) | 85.2 | -1.6(0.7) | **0.00** |
| . working memory | . Digit Span | 25.9 | -0.8(0.9) | 6.7 | -0.2(0.7) | 37.0 | -1.1(1.0)**^h^** | **0.00** |
| . conceptualization | . Similarities (WAIS-III) | 11.8 | 0.04(1.0) | 0 | 0.5(0.7) | 18.5 | -0.3(1.0) | **0.00** |
| . set activation | . Phonological Fluency (P,M,R) | 68.7 | -1.3(1.0) | 44.8 | -0.8(1.0) | 83.0 | -1.7(0.9)**^h,i^** | **0.00** |
| . set shifting | . TMT (B-A) | 85.9 | -2.9(1.8) | 70.4 | -1.7(1.2) | 95.5 | -3.6(1.8) | **0.00** |
| . set maintenance | . Odd Man Out Test  Trial 1  Trial 2  Trial 3  Trial 4  Trial 5  Trial 6  Trial 7  Trial 8  Trial 9  Trial 10  Total | 55.2 | -1.8(2.6)  -0.6(1.6)  -0.9(1.8)  -1.3(1.7)  -1.4(1.9)  -1.4(1.6)  -2.4(2.1)  -1.5(1.4)  -2.9(3.9)  -1.4(1.7)  -1.6(1.8) | 20.0 | 0.4(1.0)  0.8(0.6)  0.5(1.3)  -0.1(1.4)  -0.0(1.6)  -0.3(1.6)  -0.8(2.2)  -0.7(1.5)  0.7(2.5)  -0.2(1.2)  0.0(1.2) | 76.2 | -3.2(2.4)  -1.4(1.5)  -1.8(1.4)  -1.9(1.5)  -2.2(1.5)  -2.1(1.3)  -3.3(1.3)  -2.0(1.0)  -5.0(3.0)  -2.2(1.3)  -2.5(1.4)**^h^** | **0.00**  **0.00**  **0.00**  **0.00**  **0.00**  **0.00**  **0.00**  **0.00**  **0.00**  **0.00**  **0.00** |
| . behavioral control | . Prehension Behavior (FAB) | 30.1 | -0.8(1.1) | 16.7 | -0.4(0.9) | 38.5 | -1.0(1.2) | **0.05** |
| 1. Memory**^a^** | . RAVLT (Total) | 68.7 | -1.5(1.2)**^c^** | 34.5 | -0.6(0.9) | 86.8 | -2.1(1.1)**^c^** | **0.00** |
| - 1. Memory (**only** subcortical-frontal component) | . RAVLT (Total component) | 15.7 | -1.8(0.8) | 10.3 | -1.3(0.3) | 17.0 | -2.0(0.9) | 0.15 |
|  | . Total learning | 100 | -2.5(1.0) | 100 | -1.5(0.1) | 100 | -2.8(1.0)**^j^** | **0.02** |
|  | . Learning over trials index | 46.2 | -1.2(1.0) | 66.7 | -1.2(0.5) | 44.4 | -1.3(1.2) | 0.73 |
| Cortical features |  |  |  |  |  |  |  |  |
| - 1. Memory (**only** mediotemporal component) | . RAVLT (Total component) | 18.1 | -1.9(0.6) | 20.7 | -1.7(0.5) | 17.0 | -2.0(0.7) | 0.53 |
|  | . Long term percent retention index | 80.0 | -2.1(1.5) | 83.3 | -1.9(1.2) | 77.8 | -2.3(1.7) | 0.86 |
|  | . Delayed recognition | 60.0 | -1.6(0.8) | 50.0 | -1.5(0.9) | 66.7 | -1.6(0.7) | 0.96 |
| - 1. Memory (**both** components impaired)**^b^** | . RAVLT (Total component) | 44.6 | -2.5(0.7) | 17.2 | -2.0(0.6) | 60.4 | -2.6(0.7) | 0.07 |
|  | . Total learning | 100 | -2.9(0.8) | 100 | -1.9(0.2) | 100 | -3.1(0.8) | **0.00** |
|  | . Learning over trials index | 67.6 | -1.4(1.1) | 40.0 | -1.3(1.6) | 71.9 | -1.5(1.0)**^k^** | 0.37 |
|  | . Long term percent retention index | 89.2 | -3.2(1.3) | 80.0 | -2.5(1.3) | 90.6 | -3.3(1.3) | 0.14 |
|  | . Delayed recognition | 100 | -2.6(0.8) | 100 | -2.1(0.4) | 100 | -2.6(0.9)**^k^** | 0.23 |
| 1. Instrumental function |  | 55.6 | -1.3(1.1) | 20.7 | -0.4(0.8) | 76.5 | -1.8(1.0)**^c^** | **0.00** |
| . language | . Boston Naming Test | 11.1 | -0.2(0.9) | 6.9 | 0.2(1.0) | 13.7 | -0.4(0.8) | **0.01** |
| . visuo-constructive | . Copy of the Clock | 60.8 | -2.5(2.9) | 24.1 | -0.3(1.7) | 83.7 | -3.9(2.6) | **0.00** |
| . visuospatial | . Benton Line Orientation Test | 67.1 | -1.3(1.0) | 39.3 | -0.7(1.1) | 85.1 | -1.7(0.8)**^l^** | **0.00** |
| . visuoperceptive | . Benton Face Recognition Test | 61.1 | -1.3(1.0) | 40.7 | -0.8(1.1) | 73.3 | -1.5(0.8)**^l^** | **0.00** |
| Single domain |  | 1.2 |  | 3.3 |  | 0 |  |  |
| Multiple domain |  | 92.4 |  | 80.0 |  | 100 |  |  |

Table S-1 (Supplementary material). Cognitive performance of LSPD patients

Sub-groups were compared using the Mann-Whitney U and Wilcoxon tests (p<0.05 is significant).

LSPD-NoD Late-Stage Parkinson’s Disease patients without dementia

LSPD-D Late-Stage Parkinson’s Disease patients with dementia

MDS PDD- Parkinson’s Disease dementia criteria recommended by Movement Disorder Society Task Force

MMSE- Mini-Mental State Exam

TMT (A and B)- Trail Making Test A and B

WAIS-III- Wechsler Adult Intelligence Scale 3^rd^ Edition

FAB- Frontal Assessment Battery

RAVLT- Rey Auditory and Verbal Learning Test

^a^ Cognitive domain not considered as subcortical feature and for subcortical composite z-score calculation

^b^ Cognitive domain not considered as cortical feature and for cortical composite z-score calculation

**^c^** p<0.05, different from executive function

**^d^** p<0.05, different from orientation

**^e^** p<0.05, different from delayed recall

**^f^** p<0.05, different from orientation to place

**^g^** p<0.05, different from writing

**^h^** p<0.05, different from set shifting

**^i^** p<0.05, different from working memory

**^j^** p<0.05, different from learning over trials index

**^k^** p<0.05, different from long-term percent retention index

**^l^** p<0.05, different from visuo-constructive
