# Supplementary material for: Profile of cognitive impairment in late‐stage Parkinson's disease
Source: Brain Behav. 2022 Mar 7;12(4):e2537. doi: 10.1002/brb3.2537 (PMC9014988; doi:10.1002/brb3.2537)
Supplement: Supplementary file 2 — TABLE S2. Comparative analysis of the LSPD patients’ cognitive performance according to their cognitive profile in nondementia vs dementia groups [file BRB3-12-e2537-s002.docx]

|  | **LSPD-NoD**  **(N=30)**  **Mean (SD)** | | | | | | | | | **LSPD-D**  **(N=54)**  **Mean (SD)** | | | | | | |
| --- | --- | --- | --- | --- | --- | --- | --- | --- | --- | --- | --- | --- | --- | --- | --- | --- |
| **Clinical and cognitive description** | **Heterogeneous profile**  **(N=22)** | | **Subcortical profile**  **(N=2)** | | **Cortical profile**  **(N=2)** | | **Global impair profile**  **(N=4)** | | **p** | **Subcortical profile**  **(N=13)** | | **Cortical profile**  **(N=4)** | | **Global impair profile**  **(N=37)** | | **p** |
|  | Impair  (%) | z-score  Mean (SD) | Impair  (%) | z-score  Mean (SD) | Impair  (%) | z-score  Mean (SD) | Impair  (%) | z-score  Mean (SD) |  | Impair  (%) | z-score  Mean (SD) | Impair  (%) | z-score  Mean (SD) | Impair  (%) | z-score  Mean (SD) |  |
| Gender (M/F) | 7/15 |  | 1/1 |  | 0/2 |  | 1/3 |  | 0.72 | 6/7 |  | 1/3 |  | 20/17 |  | 0.52 |
| Age |  | 76.8(5.1) |  | 73.5(0.7) |  | 73.0(7.1) |  | 73.0(6.1) | 0.47 |  | 74.1(7.7) |  | 70.0(16.1) |  | 76.2(6.4) | 0.61 |
| Education |  | 5.5(4.1) |  | 5.0(1.4) |  | 3.5(0.7) |  | 8.3(7.7) | 0.55 |  | 7.9(4.6) |  | 7.0(4.8) |  | 6.8(4.6) | 0.74 |
| Disease duration (yrs) |  | 17.5(7.8) |  | 15.0(5.7) |  | 9.0(1.4) |  | 18.5(9.1) | 0.26 |  | 18.8(8.3) |  | 18.0(11.9) |  | 16.2(6.9) | 0.66 |
| Age at onset (yrs) |  | 59.3 (11.1) |  | 58.5(5.0) |  | 64.0(8.5) |  | 54.5(8.2) | 0.61 |  | 55.3 (10.5) |  | 52.0 (26.6) |  | 60.0(9.5) | 0.48 |
| MDS_UPDRS 1 |  | 16.0(5.5) |  | 25.5 (10.6) |  | 15.0(8.5) |  | 20.0(6.9) | 0.33 |  | 23.5(5.7) |  | 19.0(3.7) |  | 22.7(6.9) | 0.28 |
| MDS_UPDRS 2 |  | 30.0(8.7) |  | 34.0(11.3) |  | 17.5(5.0) |  | 33.5(7.2) | 0.20 |  | 33.8(8.0) |  | 32.0(14.4) |  | 37.1(9.2) | 0.34 |
| MDS_UPDRS 2.7 |  | 3.1(0.9) |  | 2.5(2.1) |  | 2.0(0.0) |  | 3.3(1.0) | 0.37 |  | 3.3(0.9) |  | 3.3(1.5) |  | 3.5(0.8) | 0.83 |
| MDS_UPDRS 2.10 |  | 1.4(1.2) |  | 0.0(0.0) |  | 0.5(0.7) |  | 0.3(0.5) | 0.10 |  | 0.9(0.8) |  | 1.5(1.3) |  | 1.0(1.4) | 0.51 |
| MDS_UPDRS 2.13 |  | 2.2(1.6) |  | 3.5(0.7) |  | 2.0(2.8) |  | 3.5(1.0) | 0.32 |  | 2.6(1.2) |  | 3.0(2.0) |  | 2.7(1.5) | 0.56 |
| MDS_UPDRS 3 |  | 51.5 (14.0) |  | 66.5 (26.1) |  | 34.0(7.1) |  | 58.0 (16.8) | 0.13 |  | 57.5 (14.9) |  | 66.5(31.0) |  | 64.8(14.5) | 0.25 |
| MDS_UPDRS 4 |  | 3.9(4.4) |  | 9.0(2.8) |  | 4.5(6.4) |  | 8.0(4.3) | 0.18 |  | 6.4(4.6) |  | 5.5(6.4) |  | 4.5(3.9) | 0.44 |
| Levodopa (% yes) | 100 |  | 100 |  | 100 |  | 100 |  | 1.00 | 100 |  | 50.0 |  | 100 |  | **0.00** |
| Hoehn & Yahr stage (0/5) |  | 3.8(0.9) |  | 3.5(2.1) |  | 4.0(0.0) |  | 3.9(1.0) | 0,99 |  | 4.0(1.1) |  | 4.3(1.0) |  | 4.3(0.9) | 0.57 |
| Schwab & England (0/100%) |  | 44.6 (14.4) |  | 40.0 (28.3) |  | 49.0(0.0) |  | 40.0 (14.1) | 0.34 |  | 32.3(9.3) |  | 37.5(15.0) |  | 31.6(10.9) | 0.58 |
| NPI delusions (0/12) (Cutoff ≥3) | 4.5 | 0.2(0.9) | 50.0 | 4.5(6.4) | 0 | 0 | 25.0 | 1.5(3.0) | 0.11 | 15.4 | 0.9(1.5) | 25.0 | 2.0(4.0) | 25.7 | 2.0(3.0) | 0.48 |
| NPI hallucinations (0/12) (Cutoff ≥3) | 4.5 | 0.3(1.3) | 50.0 | 4.5(6.4) | 0 | 1.0(1.4) | 25.0 | 1.8(2.9) | 0.10 | 30.8 | 1.9(2.3) | 0 | 0 | 40.0 | 2.5(3.2) | 0.15 |
| NPI depression (0/12) (Cutoff ≥3) | 40.9 | 2.8(2.4) | 100 | 6.0(4.2) | 0 | 1.0(0.0) | 25.0 | 2.8(2.2) | 0.27 | 84.6 | 3.8(1.6) | 50.0 | 3.3(3.6) | 65.7 | 3.6(2.4) | 0.84 |
| NPI apathy (0/12) (Cutoff ≥3) | 27.3 | 1.8(2.6) | 50.0 | 7.0(7.1) | 0 | 0 | 50.0 | 3.5(3.0) | 0.11 | 61.5 | 3.7(3.0) | 50.0 | 3.5(3.3) | 68.6 | 4.9(3.9) | 0.65 |
| NPI sleep disorders (0/12) (Cutoff ≥3) | 27.3 | 1.9(2.5) | 100 | 6.5(3.5) | 0 | 1.0(1.4) | 0 | 0.3(0.5) | 0.11 | 61.5 | 4.0(3.1) | 0 | 0.5(1.0) | 40.0 | 2.9(3.9) | 0.08 |
| Pill questionnaire (0/3) (Cutoff ≥2) | 50.0 | 1.4(1.1) | 100 | 2.5(0.7) | 0 | 1.0(0.0) | 25.0 | 1.5(1.0) | 0.23 | 76.9 | 2.2(1.1) | 75.0 | 1.8(0.5) | 97.2 | 2.8(0.6)**^b,c^** | **0.00** |
| GDS score (0/30) (Cutoff 11-20: mild depression; 21-30: severe depression) | 76.2 | 13.5(5.3) | 100 | 18.0(0.0) | 50.0 | 9.0(1.4) | 100 | 17.0(5.4) | 0.27 | 91.7 | 19.3(6.7) | 100 | 14.3(3.1) | 80.0 | 15.7(5.2) | 0.12 |
| MMSE score | 18.2 | -0.4(1.3) | 0 | -0.7(1.2) | 0 | 0.7(1.6) | 75.0 | -1.4(0.7) | 0.19 | 100 | -5.0(4.3)**^d^** | 100 | -3.1(1.2) | 100 | -7.5(2.8)**^b,c,e^** | **0.00** |
| Orientation | 9.1 | -0.1(0.9) | 100 | -2.8(1.3)**^a^** | 0 | 0.6(0.0) | 50.0 | -1.2(1.4) | **0.04** | 92.3 | -3.7(2.9) | 50.0 | -1.5(1.5) | 86.5 | -5.7(3.6)**^c,e^** | **0.03** |
| Orientation to time | 40.9 | -0.7(1.5) | 100 | -5.3(2.4) | 0 | 0.4(0.1) | 75.0 | -1.9(2.0) | 0.08 | 100 | -4.4(2.8)**^f^** | 75.0 | -2.2(1.8) | 94.6 | -6.3(3.7)**^e,f^** | 0.08 |
| Orientation to place | 4.5 | 0.4(0.4) | 0 | 0.4(0.1) | 0 | 0.5(0.1) | 25.0 | -0.3(1.4) | 0.69 | 46.2 | -1.8(3.0) | 25.0 | -0.6(1.6) | 51.4 | -2.1(2.7) | 0.64 |
| Immediate recall | 0 | 0.0(0.0) | 0 | 0 | 0 | 0.0(0.0) | 0 | 0.0(0.0) | 1.00 | 0 | -0.1(0.3) | 0 | 0 | 13.5 | -0.6(1.6) | 0.40 |
| Attention and calculation | 22.7 | -0.5(1.4) | 50.0 | -1.2(2.6) | 50.0 | -0.3(1.9) | 25.0 | -0.6(1.6) | 0.97 | 69.2 | -3.4(4.7) | 50.0 | -0.6(1.1) | 78.4 | -4.0(4.6)**^g,i^** | 0.19 |
| Delayed recall | 0 | 0.5(0.6) | 0 | 0.7(0.4) | 0 | 1.2(0.4) | 0 | 0.0(0.8) | 0.20 | 30.8 | -0.4(1.4) | 25.0 | -0.3(1.0) | 59.5 | -1.5(1.1)**^b,e,g^** | **0.00** |
| Language | 18.2 | -0.5(1.4) | 0 | 0.7(0.2) | 0 | 0.5(0.5) | 50.0 | -0.7(1.5) | 0.32 | 53.8 | -3.1(4.8) | 50.0 | -3.8(4.3) | 94.6 | -4.1(2.3)**^e,g^** | 0.09 |
| Naming | 0 | 0 | 0 | 0 | 0 | 0 | 0 | 0 | 1.00 | 0 | 0 | 0 | 0 | 0 | -0.1(0.3) | 0.49 |
| Repetition | 0 | 0 | 0 | 0 | 0 | 0 | 0 | 0 | 1.00 | 0 | 0 | 0 | 0 | 35.1 | -0.4(0.5)**^b^** | **0.02** |
| Verbal complex order comprehension | 36.4 | -1.4(2.7) | 0 | 0.4(0.1) | 50.0 | -0.5(1.3) | 50.0 | -0.8(1.3) | 0.41 | 53.8 | -3.9(5.8) | 75.0 | -4.5(4.6) | 81.1 | -3.5(2.7)**^e^** | 0.73 |
| Reading comprehension | 0 | 0.1(0.2) | 0 | 0 | 0 | 0.2(0.2) | 0 | 0 | 0.45 | 0 | 0.0(0.4) | 0 | 0.1(0.2) | 13.5 | -1.1(3.2)**^h^** | 0.83 |
| Writing | 0 | 0.3(0.3) | 0 | 0.5(0.2) | 0 | 1.0(0.6) | 50.0 | -0.6(1.0)**^c^** | **0.03** | 23.1 | -0.5(1.7) | 50.0 | -1.4(2.6) | 64.9 | -1.3(1.4)**^h^** | 0.17 |
| Construction | 13.6 | 0.4(1.1) | 50.0 | -0.6(2.0) | 50.0 | 0.1(1.8) | 50.0 | -0.6(1.3) | 0.28 | 53.8 | -1.0(1.2) | 75.0 | -2.0(0.8) | 70.3 | -1.4(0.9)**^g^** | 0.30 |
| Executive function | 0 | -0.3(0.4) | 50.0 | -0.9(0.2) | 0 | -0.3(0.2) | 25.0 | -1.0(0.7)**^a^** | **0.03** | 76.9 | -1.1(0.4)**^j^** | 25.0 | -0.5(0.5) | 94.6 | -1.8(0.6)**^b,c,e^** | **0.00** |
| Working memory | 9.1 | -0.2(0.7) | 0 | -0.3(1.1) | 0 | -0.3(0.4) | 0 | -0.6(0.3) | 0.51 | 46.2 | -1.0(1.0) | 0 | 0.4(1.0)**^b^** | 37.8 | -1.2(0.9)**^c,k^** | **0.03** |
| Conceptualization | 0 | 0.6(0.8) | 0 | 0.7(0.5) | 0 | 0.2(0.2) | 0 | 0.4(0.5) | 0.76 | 23.1 | -0.1(1.2) | 0 | 0.2(0.9) | 18.9 | -0.4(0.9) | 0.31 |
| Set activation | 38.1 | -0.6(1.1) | 50.0 | -1.2(0.4) | 0 | -0.5(0.3) | 100 | -1.5(0.4) | 0.23 | 69.2 | -1.4(0.8)**^k^** | 75.0 | -1.5(0.8) | 88.9 | -1.8(1.0)**^k^** | 0.35 |
| Set shifting | 65.0 | -1.4(0.9) | 100 | -2.9(0.4) | 100 | -2.1(0.0) | 75.0 | -2.8(1.9) | 0.08 | 100 | -2.7(0.7) | 100 | -1.5(0.1) | 93.8 | -4.0(1.9)**^c^** | **0.02** |
| Set maintenance | 10.5 | 0.4(0.8) | 100 | -3.0(0.0) | 0 | 0.4(0.0) | 50.0 | -1.0(1.3) | 0.06 | 22.2 | -0.9(1.1) | 66.7 | -1.5(2.4) | 93.3 | -3.1(0.9)**^b,e,k^** | **0.00** |
| Behavioral control | 18.2 | -0.5(0.9) | 0 | 0 | 0 | 0 | 25.0 | -0.5(1.0) | 0.72 | 33.3 | -0.8(1.1) | 0 | 0 | 44.4 | -1.1(1.3)**^k^** | 0.20 |
| Memory | 28.6 | -0.4(0.8) | 50.0 | -0.7(0.4) | 0 | -0.4(0.3) | 75.0 | -1.7(1.2) | 0.22 | 69.2 | -1.3(1.2) | 50.0 | -1.1(0.5) | 97.2 | -2.4(0.8)**^b,c,l^** | **0.00** |
| Memory (only subcortical-frontal component) | 9.5 | -1.2(0.1) | 50.0 | -1.6(0.0) | 0 | 0 | 0 | 0 | 0.67 | 53.8 | -2.2(1.0) | 0 | 0 | 5.6 | -1.5(0.5) | 0.50 |
| . Total learning | 100 | -1.4(0.1) | 100 | -1.6(0.0) | 0 | 0 | 0 | 0 | 0.67 | 100 | -2.8(1.1) | 0 | 0 | 100 | -2.8(1.1) | 1.0 |
| . Learning over trials | 50 | -0.9(0.4) | 100 | -1.7(0.0) | 0 | 0 | 0 | 0 | 0.67 | 57.1 | -1.6(1.2) | 0 | 0 | 0 | -0.3(0.1) | 0.06 |
| Memory (only mediotemporal component) | 14.3 | -2.0(0.6) | 50.0 | -1.1(0.0) | 50.0 | -1.8(0.0) | 25.0 | -1.5(0.0) | 0.47 | 7.7 | -1.6(0.0) | 100 | -1.7(0.8) | 11.1 | -2.3(0.7) | 0.32 |
| . Long term percent retention index | 100 | -2.6(1.2) | 100 | -1.6(0.0) | 100 | -1.6(0.0) | 0 | -0.4(0.0) | 0.22 | 100 | -1.1(0.0) | 75.0 | -2.3(2.0) | 75.0 | -2.6(1.7) | 0.85 |
| . Delayed recognition | 33.3 | -1.3(1.0) | 0 | -0.5(0.0) | 100 | -2.0(0.0) | 100 | -2.5(0.0) | 0.47 | 100 | -2.0(0.0) | 25.0 | -1.1(0.6) | 100 | -2.0(0.4) | 0.16 |
| Memory (both components impaired) | 14.3 | -1.6(0.2) | 0 | 0 | 0 | 0 | 50.0 | -2.6(0.5) | 0.20 | 23.1 | -1.7(0.1) | 0 | 0 | 80.6 | -2.7(0.6) | **0.01** |
| . Total learning | 100 | -1.8(0.1) | 0 | 0 | 0 | 0 | 100 | -2.0(0.4) | 1.0 | 100 | -2.1(0.2) | 0 | 0 | 100 | -3.2(0.7) | **0.03** |
| . Learning over trials | 33.3 | -0.7(0.7) | 0 | 0 | 0 | 0 | 50.0 | -2.3(2.4) | 0.40 | 66.7 | -1.0(0.2) | 0 | 0 | 72.4 | -1.5(1.0)**^m^** | 0.38 |
| . Long term percent retention index | 66.7 | -1.7(0.8) | 0 | 0 | 0 | 0 | 100 | -3.7(0.4) | 0.20 | 100 | -1.7(0.2) | 0 | 0 | 89.7 | -3.4(1.2) | **0.04** |
| . Delayed recognition | 100 | -2.0(0.5) | 0 | 0 | 0 | 0 | 100 | -2.3(0.4) | 0.80 | 100 | -1.8(0.3) | 0 | 0 | 100 | -2.7(0.9)**^m^** | **0.04** |
| Instrumental function | 9.5 | -0.2(0.6) | 0 | -0.1(0.2) | 100 | -1.3(0.3) | 50.0 | -1.4(1.0)**^a^** | **0.02** | 16.7 | -0.6(0.5) | 75.0 | -1.8(1.1) | 97.2 | -2.3(0.7)**^b,l^** | **0.00** |
| Language | 4.5 | 0.4(1.0) | 0 | 0.5(0.0) | 0 | -1.0(0.0) | 25.0 | -0.8(0.9) | 0.05 | 0 | 0.2(0.8) | 50.0 | -1.1(1.0)**^b^** | 14.3 | -0.5(0.7) | **0.02** |
| Visuo-constructive | 23.8 | -0.1(1.5) | 0 | 0.7(0.6) | 50.0 | -0.6(1.3) | 25.0 | -1.3(3.2) | 0.47 | 40.0 | -0.5(1.3) | 100 | -4.3(2.0) | 94.3 | -4.9(2.1)**^b,e^** | **0.00** |
| Visuospatial | 28.6 | -0.5(1.1) | 50.0 | -1.0(1.4) | 100 | -1.5(0.0) | 75.0 | -1.4(0.9) | 0.53 | 60.0 | -1.4(0.7) | 75.0 | -0.5(1.9) | 94.1 | -1.9(0.4)**^b,n^** | **0.00** |
| Visuoperceptive | 30.0 | -0.5(1.1) | 0 | -0.5(0.0) | 100 | -2.0(0.0) | 100 | -2.0(0.0)**^a^** | **0.04** | 37.5 | -0.8(1.1) | 75.0 | -1.4(0.9) | 82.4 | -1.7(0.6)**^b,n^** | **0.04** |

Table S-2 (Supplementary material). Comparative analysis of the LSPD patients’ cognitive performance according to their cognitive profile in non-dementia vs dementia groups.

Sub-groups were compared using the Chi-squared (nominal variables), Wilcoxon, and the Kruskal-Wallis tests with post hoc pairwise analysis and Mann-Whitney U test for pairwise comparison between non-dementia and dementia sub-groups (continuous variables) (p<0.05 is significant)

LSPD- Late-Stage Parkinson’s disease patients

NPI- Neuropsychiatric Inventory

GDS- Geriatric Depression Scale

MMSE- Mini-Mental State Exam

^a^ p<0.05, different from heterogeneous group, with Bonferroni correction

^b^ p<0.05, different from subcortical profile group, with Bonferroni correction

^c^ p<0.05, different from cortical group, with Bonferroni correction

^d^ p<0.05, different from subcortical profile non-demented group

^e^ p<0.05, different from global impairment non-demented group

^f^ p<0.05, different from orientation to place

^g^ p<0.05, different from orientation

^h^ p<0.05, different from verbal complex order comprehension

^i^ p<0.05, different from delayed recall

^j^ p<0.05, different from instrumental function

^k^ p<0.05, different from set shifting

^l^ p<0.05, different from executive function

^m^ p<0.05, different from long-term percent retention index

^n^ p<0.05, different from visuo-constructive
